# Supplementary material for: Single Cell Transcriptomic and Chromatin Profiles Suggest Layer Vb Is the Only Layer With Shared Excitatory Cell Types in the Medial and Lateral Entorhinal Cortex
Source: Front Neural Circuits. 2022 Jan 26;15:806154. doi: 10.3389/fncir.2021.806154 (PMC8826650; doi:10.3389/fncir.2021.806154)
Supplement: Supplementary file 14 [file Data_Sheet_1.ZIP › Sup_vs_Deep_Motifs/homerResults/motif4.similar.html]

motif4

## Information for motif4

C
A
T
G
G
T
A
C
G
C
A
T
T
C
G
A
G
C
T
A
C
G
A
T
A
G
C
T
C
G
T
A
C
G
T
A
C
T
A
G
C
T
A
G
G
C
A
T
  
Reverse Opposite:  

C
G
T
A
G
A
T
C
G
A
T
C
G
C
A
T
G
C
A
T
T
C
G
A
G
C
T
A
C
G
A
T
A
G
C
T
C
G
T
A
C
A
T
G
G
T
A
C
  

|  |  |
| --- | --- |
| p-value: | 1e-135 |
| log p-value: | -3.126e+02 |
| Information Content per bp: | 1.609 |
| Number of Target Sequences with motif | 2745.0 |
| Percentage of Target Sequences with motif | 8.19% |
| Number of Background Sequences with motif | 425.7 |
| Percentage of Background Sequences with motif | 4.99% |
| Average Position of motif in Targets | 309.9 +/- 230.9bp |
| Average Position of motif in Background | 360.7 +/- 262.9bp |
| Strand Bias (log2 ratio + to - strand density) | -0.1 |
| Multiplicity (# of sites on avg that occur together) | 1.05 |
| Motif File: | file (matrix) reverse opposite |

### Similar de novo motifs found

|  |  |  |  |  |  |  |  |
| --- | --- | --- | --- | --- | --- | --- | --- |
| Rank | Match Score | Redundant Motif | P-value | log P-value | % of Targets | % of Background | Motif file |
| 1 | 0.738 | C G A T G C T A G C T A C G A T C G A T G C T A G C T A C G A T C G A T C G T A C T A G G T A C | 1e-103 | -238.809009 | 9.77% | 6.64% | motif file (matrix) |
| 2 | 0.660 | C G A T C T G A C T G A A C G T C T A G T C G A T A C G A G T C | 1e-94 | -217.082364 | 16.58% | 12.69% | motif file (matrix) |
